# First Complete Genome Sequence of Genotype III Japanese Encephalitis Virus Isolated from a Stillborn Piglet in India

**DOI:** 10.1128/genomeA.01503-16

**Published:** 2017-01-19

**Authors:** P. A. Desingu, Pradeep K. Ray, Jeny K. John, T. Das, Z. B. Dubal, K. K. Rajak, R. K. Singh, G. Saikumar

**Affiliations:** a Division of Pathology, IVRI, Izatnagar, Uttar Pradesh, India; b Division of Veterinary Public Health, IVRI, Izatnagar, Uttar Pradesh, India; c Division of Biological Products, IVRI, Izatnagar, Uttar Pradesh, India; d Director, IVRI, Izatnagar, Uttar Pradesh, India

## Abstract

We report here the first complete genome of the Japanese encephalitis virus (JEV) genotype III strain JEV/SW/IVRI/395A/2014, isolated from stillborn piglets in India. It shares 99% identity with strain JaOArS982 and a few other strains from Japan.

## GENOME ANNOUNCEMENT

Japanese encephalitis virus (JEV) is a mosquito-borne, zoonotic, viral disease caused by a *Flavivirus*. It is estimated to infect about 68,000 children and causes 10,000 to 15,000 deaths per year in Asia (1, 2). It also causes reproductive failure in pigs (3, 4). In India, most of the JEV studies in pigs have been limited to assessing the seroprevalence in endemic areas. Recently, we characterized a JEV isolated from stillborn piglets based on E and PrM proteins (4). Here, we report the complete genome sequence of strain JEV/SW/IVRI/395A/2014, isolated from stillborn piglets.

Viral RNA of JEV/SW/IVRI/395A/2014 was isolated from a stillborn piglet’s brain tissue using Vero cells per standard procedure (4). Published and self-designed primers were used for amplification of overlapping fragments of the JEV genome (5, 6). Cloning and sequencing were carried out per standard procedure (4).

The raw sequence data obtained were assembled using MEGA6 software. The complete genome of this isolate has 10,976 nucleotides (nt) with 95 nt at the 5′ untranslated region (UTR), as well as a 10,296-nt open reading frame (single) corresponding to 3,432 amino acids and 582 nt of the 3′ UTR. The NCBI BLAST analysis revealed that this isolate shared 99% identity with strains JaOArS982 and JaTAn1/75 (swine serum) of Japan, 98% identity with JEV vaccine strains SA14, SA14-14-2, and SA14-12-1-7, and 97% identity with the Indian JEV strain GP78. The sequence alignments and phylogenetic analysis revealed that the virus belongs to genotype III. This is the first report of a complete genome sequence of JEV isolated from a stillborn piglet in India. It shares close identity with JEV vaccine strains and hence may be a promising candidate virus for developing a vaccine for use in pigs.

### Accession number(s).

The complete genome sequence of the JEV/SW/IVRI/395A/2014 isolate has been deposited in NCBI GenBank under the accession number KP164498.
